# The inverted pyramid of care in modern-day obstetrics

**DOI:** 10.1371/journal.pmed.1005255

**Published:** 2026-09-28

**Authors:** Laura A. Magee, Peter von Dadelszen, Kypros H. Nicolaides

**Affiliations:** 1 Department of Women and Children’s Health, School of Life Course and Population Health Sciences, Faculty of Life Sciences & Medicine, King’s College London, London, United Kingdom; 2 Fetal Medicine Research Institute, King’s College Hospital, London, United Kingdom

## Abstract

- Traditional antenatal care consists of an accelerating frequency of visits with advancing gestational age, as the bulk of pregnancy complications occur in late pregnancy.
- The ‘inverted pyramid of care’ concept describes a focus on structured risk assessment in early pregnancy, to facilitate prevention and enhanced surveillance of those at increased risk of maternal, intrinsic fetal, and uteroplacental problems.
- Since publication of this concept in 2011, it has become clear that early pregnancy screening may be contingent, with a sub-population identified for re-screening in mid- or late pregnancy.
- Maternal conditions (such as pre-eclampsia), intrinsic fetal conditions (such as chromosomal abnormalities) and uteroplacental problems (such as placenta accreta spectrum) can now be predicted or identified at earlier stages.
- This Policy Forum advocates for building risk-stratified care pathways around early prediction, prevention, and personalised surveillance. We provide updated guidance for ‘inverted’ antenatal care, including optimal screening timepoints and the most accurate methods of screening.
- We also highlight recent advances in diagnostics and treatment that we anticipate will further improve antenatal care in the future, including development and integration of artificial intelligence-assisted technologies.

## Abstract 2

In this Policy Forum, Laura Magee and colleagues provide updated guidance for ‘inverted’ antenatal care, including optimal screening timepoints and the most accurate methods of screening. They also highlight recent advances in diagnostics and treatment, including development and integration of artificial intelligence-assisted technologies.

## Introduction

Before 1900, maternity care focused on attendance at birth of the wealthy, by those of variable skill. Maternal mortality was very high (400–500 per 100,000 births) and this continued into the 20th century [1]. In 1929, the UK Government introduced universal antenatal care, based on timely detection and intervention for maternal and fetal complications [2]. Universal antenatal care was based on visit frequency, with more visits in late pregnancy when complications most commonly occur. Maternal mortality—due largely to sepsis, haemorrhage, and pre-eclampsia—declined rapidly during the 1930s, reaching <100 per 100,000 births by the 1950s, and then <10 per 100,000 live births by the late 20th century. This principle of universal antenatal care remains the standard globally, even when the total number of visits has been reduced [3]. In contrast, the reduction in perinatal mortality—driven largely by congenital anomalies, prematurity, and intrapartum complications—was more gradual, related to developments in antenatal surveillance, birth attendance in hospital, pre-eclampsia management, and neonatal unit care [4].

The concept of the ‘inverted pyramid of care’ was introduced in 2011 [5], to describe care that has a focus not only on timely diagnosis and treatment of complications, but also on early risk stratification, available preventive therapy, and focused surveillance for increased risk (Fig 1). We (the authors) continue to advocate the principles of the original concept, building risk-stratified care pathways around early prediction, prevention, and personalised surveillance, to maximise the opportunity for intervention to improve outcomes.

**Fig 1:**
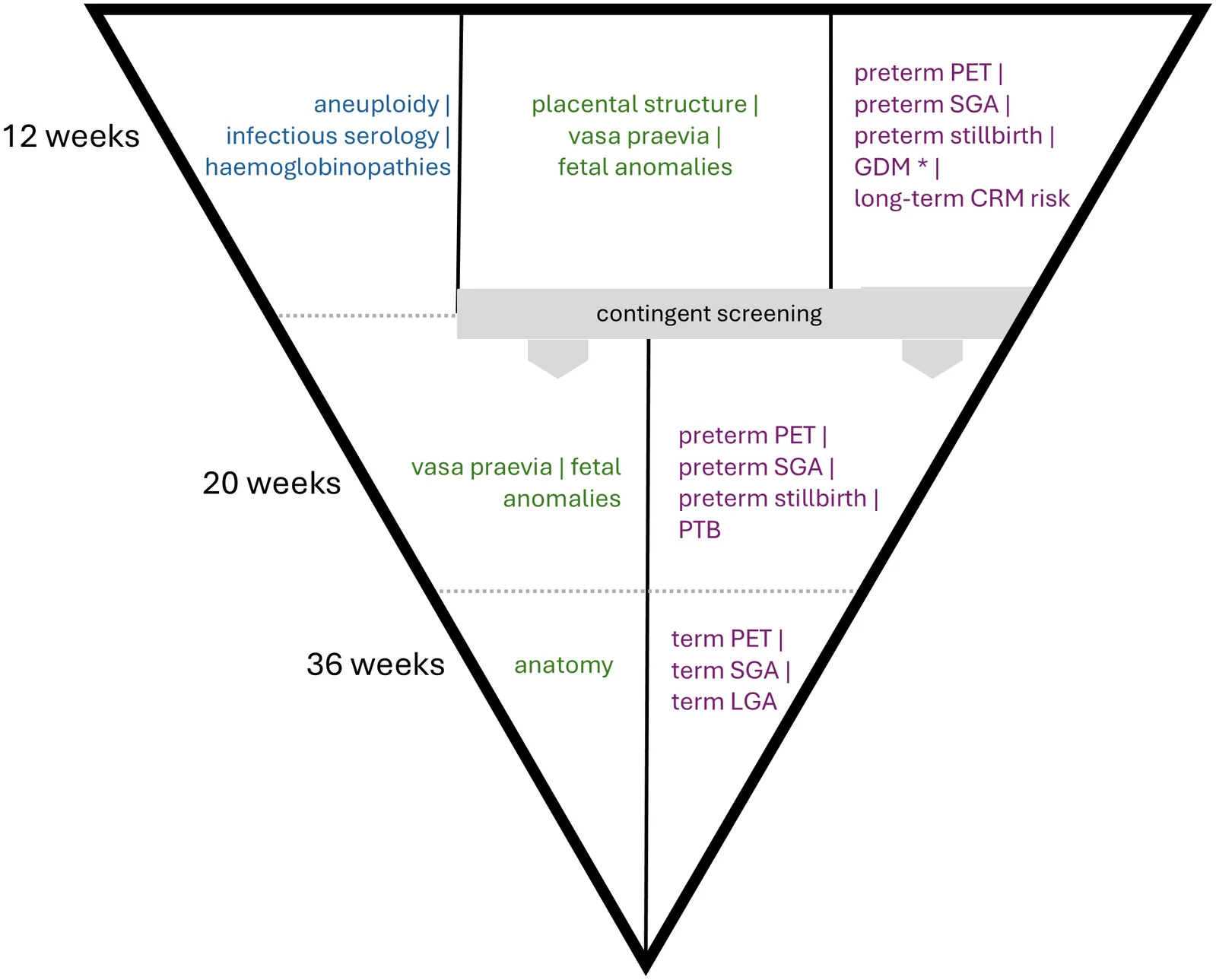
The inverted pyramid of care. This inverted pyramid of care proposes a focus on the first, rather than the third, trimester of pregnancy. The concept emphasises that many pregnancy complications can be predicted in early pregnancy, by combining maternal history, blood pressure and other biophysical measurements, and biochemical testing. CRM, cardio-renal-metabolic; GDM, gestational diabetes mellitus; LGA, large-for-gestational age; PET, pre-eclampsia; PTB, preterm birth; SGA, small-for-gestational age. * The diagnosis of GDM is usually made at 24–28 weeks’ gestation, by oral glucose tolerance testing.

In this Policy Forum, we update the ‘inverted pyramid of care’ concept, and outline optimal screening throughout the care pathway, using the most accurate methods of screening at each time point. We discuss evidence for condition-specific screening in early-, mid- or late pregnancy, with timing of screening summarised by gestational age and for relevant conditions (see Table 1). We distinguish between advances ready for implementation as well as others that are promising, but not yet policy-ready. This is an expert narrative synthesis, rather than a systematic review; evidence was identified from key trials, clinical guidelines, and targeted literature searches, prioritising the most methodologically robust and recent studies. Nevertheless, many studies come from a small number of research groups and high-resource settings, with broader independent validation still needed.

**Table 1:** Screening for adverse maternal-fetal outcomes in early-, mid- and late pregnancy.

Condition for screening	Early pregnancy (11–13 weeks)	Mid-pregnancy (19–23 weeks)	Late pregnancy (35–36 weeks)	Post-partum
Fetal chromosomal abnormalities	●			
Infectious diseases (HIV, Hepatitis B, syphilis, CMV)	●			
Haemoglobinopathy	●			
Placental structure/fetal anomalies	●	●	●	●
Placental dysfunction (PE, SGA, stillbirth)	●	●	●	
Large-for-gestational age			●	
Gestational diabetes mellitus	●^*^	●		
Preterm birth	●	●		
Cardio-renal-metabolic disease	●			●^†^

* Identification of risk may lead to early pregnancy oral glucose tolerance testing, which if normal, should be followed by testing again at 24–28 weeks.

† Risk assessment based on early pregnancy risk factors is supplemented by pregnancy outcome.

Abbreviations: CMV, cytomegalovirus; HIV, human immunodeficiency virus; PE, pre-eclampsia; SGA, small-for-gestational age.

## Designing and implementing screening programmes

A screening programme is justified when early detection of a serious condition, using an acceptable and accurate test, leads to better outcomes than usual care, at acceptable cost and harm [6]. Antenatal screening for syphilis is widely regarded as the flagship antenatal screening programme, associated with reductions of 97% in congenital syphilis, 82% in stillbirth, 64% in preterm birth, and 80% in neonatal death [7].

A screening test should have a low screen-positive rate (SPR, the proportion of the population labelled ‘positive’), high detection rate (DR, the proportion of cases that are correctly identified), and few if any harms (clinical [e.g., miscarriage], psychological [e.g., anxiety], or health system [e.g., costs]). Where to set the risk threshold for action is a complex policy decision, dependent on the seriousness of the condition, the safety and cost of confirmatory testing, the effectiveness of available interventions, and the capacity of the health system. For example, the SPR was initially set at a target of 5% for serum screening for Down syndrome, when women who screened positive were offered invasive prenatal diagnosis, which carries a small risk of miscarriage [8]. However, a higher SPR can be accepted if the confirmatory strategy is safe, such as cell-free DNA (cfDNA) testing (see next section). Important to also consider is that the performance of a screening test may vary by population and baseline risk; for example, a positive test is far less likely to be a true positive in a low-risk population.

Of note, screening incorporating biophysical and biochemical marker data requires complex management and coordinated governance, including test acquisition, laboratory processing, risk modelling, and post-test care pathways. Effective programmes integrate robust quality assurance, workforce training, data systems for audit and recalibration, and explicit strategies to manage false positives and downstream workload, ensuring that screening leads to proportionate, beneficial clinical action. For screening that involves biomarker data, values must be adjusted for baseline characteristics used to calculate prior risk. Adjustments must be ongoing, as part of a continuous quality control infrastructure, and deviations addressed through staff retraining or laboratory standards [9].

## Fetal chromosomal abnormalities and other first trimester diagnostics

Fetal chromosomal abnormalities occur in 5–7 in every 1,000 pregnancies and are most commonly trisomy (additional copy of a chromosome, commonly 21, 18, or 13), sex chromosomal defects, or triploidy (where the fetus has three complete sets of chromosomes [*N* = 69] instead of two [*N* = 46]). Several traditional approaches are used for screening. Together, nuchal translucency thickness (a measurement of the fluid-filled space behind a fetus’ neck, indicative of chromosomal abnormalities and measured by ultrasound, usually between 11 and 13 weeks), fetal heart rate, pregnancy-associated plasma protein-A levels, and free beta-human chorionic gonadotropin (a hormone blood marker), can detect ~90% of cases, at a risk cut-off of 1:300 for trisomy 21, at SPR ~5% [10].

A major breakthrough has been cfDNA testing of maternal blood; these small fragments of DNA circulate freely in plasma, outside cells, having been released mainly through cell breakdown. During pregnancy, ~10%–15% of cfDNA circulating in maternal blood originates from the placenta, and specifically from trophoblast cells undergoing apoptosis. Analysis of this ‘fetal’ fraction of cfDNA offers diagnosis of fetal chromosomal abnormalities, with DR >99% for trisomy 21, at SPR <0.1% [11]. cfDNA has been implemented for universal testing in some countries, like Belgium and the Netherlands. However, as cost per patient is high (>£200 in the UK), other countries offer contingent screening to reduce costs; those who screen positive with traditional screening, are offered cfDNA testing, achieving a DR of 96% for trisomy 21, at a risk cut-off of 1:1,000 (at SPR = 13%).

Other first trimester diagnostics in practice include serology for infectious disease (Fig 1), with the aim of reducing mother-to-child transmission of human immunodeficiency virus or Hepatitis B to <1% [12,13]. Serology is also used for the detection of haemoglobinopathies. Universal testing (e.g., haemoglobin electrophoresis) identifies maternal carriers for sickle cell, thalassaemia, and other haemoglobin variants. This enables partner testing and if both parents are carriers, genetic counselling and prenatal diagnosis. Where haemoglobinopathy prevalence is <1%, contingent screening can be undertaken for ethnicity/family origin, and then diagnostic testing offered, for detection >90% of those at risk [14].

## Placental structure, cord insertion, and fetal anomalies

Placenta accreta spectrum (PAS), with a prevalence of 1–2 in every 1,000 pregnancies [15], involves abnormally adherent or invasive placentation due to absence or deficiency of the decidua basalis (the maternal component of the placenta at the site of implantation), rather than a generalised loss of uterine wall structure [16]. Most cases are in the lower uterine segment, developing inside a prior Caesarean scar or myometrial defect/indentation. All are diagnosed at the routine 20-week scan, by at least three of five relevant ultrasound markers: (1) retroplacental myometrial thickness <1 mm; (2) retroplacental arterial-trophoblastic blood flow; (3) intra-placental lacunar spaces; (4) non-visible Caesarean scar; and (5) bladder wall interruption [17]. If PAS is diagnosed, prompt referral should be made to a regional referral centre with relevant expertise, as PAS is a leading cause of severe maternal morbidity and mortality through massive obstetric haemorrhage at birth [18].

In vasa praevia (<1 in 1,000 pregnancies [19]), fetal blood vessels are unsupported by the placenta or umbilical cord, close to the internal cervical os (the opening at the upper end of the cervical canal, where the cervix meets the lower uterine cavity), and vulnerable to rupture, which results in haemorrhagic fetal death. Associated stillbirth (~50%) and neonatal death and morbidity are largely preventable by preterm elective Caesarean [19]. All vasa praevia can be detected by contingent screening (Fig 1), offering transvaginal ultrasound at 20 weeks [13] to those with low-lying placenta or velamentous cord insertion at the inferior part of the placenta in early pregnancy, a practice that falls within what decision-makers are considered willing to pay by contemporary standards [20].

Of fetal structural abnormalities (2%–3% of pregnancies), which includes 93% of eight major anomalies [21], ~ 25% are diagnosed by first-trimester ultrasound which is cost-effective compared with no first-trimester anatomy screening [22]. A further 25% of anomalies are detected by the 20-week fetal ultrasound, and 25% by the 36-week ultrasound. While not yet widely implemented, a 36-week scan could facilitate: late termination (where legal) for anomalies with severe impairment; specialist neonatal care planning; timing and place of birth; or timely postnatal investigation and intervention before neonatal/infant illness develops. The remaining 25% of anomalies are diagnosed after birth [23].

Automated image processing may be improved across gestational age by artificial intelligence (AI), to increase the proportion of anomalies detected antenatally. AI is an emerging technology that can produce highly structured evaluations [24]. The International Society for Ultrasound in Obstetrics and Gynaecology views AI as an adjunct to improve ultrasound quality and access, not an autonomous diagnosis replacement [25]. AI-assisted image processing may be possible, even by non-experts, to improve access to anomaly scans and in particular, to identify normal anatomical structures.

## Placental dysfunction

Placental dysfunction-related outcomes include pre-eclampsia (20–40 in 1,000 pregnancies), small-for-gestational age (SGA) infants (defined by a birthweight <10th percentile; 100 in 1,000 pregnancies), and stillbirth (~4 in 1,000 births) [26]. Screening for placental dysfunction extends into mid- and late pregnancy.

### Twelve weeks’ gestation

Preterm pre-eclampsia (8 in 1,000 pregnancies) is rare, but it is the form of disease with the highest individual risks of adverse outcomes [27], particularly at <32 weeks [28,29]. Prediction of preterm pre-eclampsia is achieved using the widely-validated Fetal Medicine Foundation (FMF) competing-risks model [30–33], based on the pathophysiology of pre-eclampsia. This survival–time model incorporates a prior distribution of gestational age at delivery with pre-eclampsia, derived from maternal characteristics, with likelihood functions from biomarkers (mean arterial pressure [MAP], uterine artery Doppler, and placental growth factor [PlGF]), to estimate an individual’s risk of delivery with pre-eclampsia before a specified gestational age [9] (Table 2). Use of specialised biomarkers may limit use, even in well-resourced settings. Nevertheless, the FMF strategy is more cost-effective [34] than clinical risk factor screening, which detects fewer cases of preterm pre-eclampsia (Table 2), particularly for women of Black ethnicity [35]. An additional advantage of the competing-risks model is that detection can be increased by lowering the risk cut-off, increasing detection at a higher SPR [9]. Importantly, based on shared underlying pathophysiology, being screen-positive for preterm pre-eclampsia (at 10% SPR) identifies within that group half of infants born preterm with birthweight <10th percentile [36], and two-thirds of placental dysfunction-related stillbirth (associated with pre-eclampsia or birthweight <10th percentile [36]) before 32 weeks.

**Table 2:** Screening for placental dysfunction using the competing risks model.

	Birth at <32 weeks		Birth at <37 weeks		Birth at ≥37 weeks	
	DR	SPR^*^	DR	SPR^*^	DR	SPR^*^
**11–13 weeks**						
Pre-eclampsia	100%	10%	75%	10%	34%	10%
			90%	30%		
SGA (birthweight <10th percentile)	50%	10%	–	–	–	–
Placental dysfunction-related stillbirth	70%	10%	–	–	–	–
**19–23 weeks**						
Pre-eclampsia	90%	3%	90%	27%	–	–
SGA (birthweight <10th percentile)	80%	20%	80%	30%	–	–
Placental dysfunction-related stillbirth	73%	10% for SGA	70% for SGA	10% for SGA	–	–
**35–36 weeks**						
Pre-eclampsia	NA	NA	NA	NA	75%	10%
SGA (birthweight <10th percentile)	NA	NA	NA	NA	80%	30% (using EFW <35th %ile)
FGR (SGA with abnormal Dopplers)	NA	NA	NA	NA	80%	25% (using EFW <30th %ile)
Placental dysfunction-related stillbirth	NA	NA	NA	NA	–	–

*SPR is listed for the outcome of interest at the specified gestational age, unless otherwise stated.

Abbreviations: EFW, estimated fetal weight; DR, detection rate; FGR, fetal growth restriction; %ile, percentile; PE, pre-eclampsia; SGA, small-for-gestational age; SPR, screen-positive rate.

Among women at increased risk of preterm pre-eclampsia, low-dose aspirin (150 mg per night, from <16 weeks) reduces preterm pre-eclampsia at <32 or <37 weeks, by ~90% and ~60%, respectively [37]. This raises the possibility (yet to be proven) that complications associated with preterm pre-eclampsia may also be prevented, namely preterm SGA (without pre-eclampsia) at <32 weeks by 40% [38], and associated stillbirths at <32 weeks, most of which are also SGA.

### Twenty weeks’ gestation

Re-screening for placental dysfunction at 20 weeks identifies additional pregnancies that may benefit from enhanced surveillance until 36 weeks (Fig 1). Implementation would require, at minimum, blood pressure measurement, which is not part of the 20-week ultrasound (non-clinical) visit.

Using maternal history, MAP, and uterine artery pulsatility index, and estimated fetal weight (EFW) at 20 weeks, 90% of preterm pre-eclampsia can be detected [39] (Table 2). Enhanced surveillance may reduce adverse outcomes until 36 weeks, when all women should be screened for the risk of term pre-eclampsia. Using the same approach, 80% of preterm SGA babies can be identified among singleton pregnancies at <32 weeks (SPRs = 20%) or <36 weeks (SPRs = 30%) [40]. Research is required to evaluate whether outcomes (particularly placental dysfunction-related preterm stillbirth [41,42]) could be improved by offering additional ultrasound scans and timed birth for non-reassuring fetal status.

### Thirty-six weeks’ gestation

While clinical assessment is routine at 36 weeks [43–45], fetal ultrasound is not. ~75% of pre-eclampsia occurs at term, when it is responsible for more than half of maternal and one-third of pre-eclampsia-related perinatal complications associated with pre-eclampsia [27]. The UK clinical risk factors for pre-eclampsia detect one-third of those who will develop term pre-eclampsia (at SPR = 10%). In contrast, at 36 weeks, maternal history, MAP, PlGF, and soluble fms-like tyrosine kinase-1 (sFlt-1; a placenta-secreted protein) detect ~75% of such pregnancies across multiple settings [30] (Table 2). In the PREVENT-PE (pre-eclampsia) trial of 8,136 women, personalised pre-eclampsia risk-stratified timed birth, based on pre-eclampsia risk at 35–36 weeks, reduced the incidence of pre-eclampsia by 30%, with no difference in emergency Caesarean or neonatal unit admission ≥48 hours [46], SGA infants or stillbirths, at a cost-savings of £500 per pre-eclampsia case averted with volume-based pricing of angiogenic markers.

The vast majority (~85%) of SGA neonates are born at term, when they represent 30% of stillbirths [47]. However, prediction of fetal size at birth remains challenging. While symphysis-fundal height is widely recommended due to ease of use and low cost, it is neither sensitive nor specific for detection of poor fetal growth, and did not improve clinical outcomes over clinical palpation [48]. Ultrasonographic assessment of fetal growth is also challenged by measurement error in fetal biometry, which causes substantial error in estimated fetal weight, and misclassification of SGA fetuses. To detect at least 80% of fetuses that will be SGA, estimated fetal weight cut-offs should be <30–35th percentile (Table 2) [49]. If spread in the error distribution were halved, almost 90% of those at risk of SGA could be detected at low SPR (2%), an improvement in inter-observer variability that could be achieved by AI [50,51].

AI is well-suited to evaluating fetal size, given the highly structured evaluation requiring automated plane recognition, use of standardised anatomical planes (e.g., head or abdominal circumference), precise automated measurements, and real-time analysis of cine loops (in contrast to taking measurements from one frame that has been frozen) [24]. With expansion of the relevant workforce, AI-assisted ultrasound is likely to replace clinical symphysis-fundal height assessment of fetal size, which is inadequate to rule-in or rule-out small babies [52].

## Large-for-gestational age infants

The measurement error in fetal biometry in the third trimester cited above, results in misclassification of large-for-gestational age [LGA]) fetuses (>90th percentile for birthweight) [53]. LGA infants are at increased risk of intrapartum trauma (as is the mother), and newborn and paediatric morbidity. Notwithstanding challenges of measuring fetal biometry, labour induction at 38 weeks for EFW >95th percentile, and 39 weeks for EFW at 90–95th percentile, may achieve emergency Caesarean rates (for fetal compromise or failure to progress in labour) comparable to spontaneous labour at 40 weeks among normally-grown fetuses; these results are consistent with benefits (reduced shoulder dystocia) of timed birth at 37–38 weeks in two randomised trials [54–56]. If conservative management until 41 weeks were considered, elective Caesarean may avoid a marked increase in emergency Caesarean following labour induction at this gestational age.

## Gestational diabetes mellitus

Gestational diabetes mellitus (GDM) is the most common medical complication of pregnancy. A major focus of GDM diagnosis and management is prevention of LGA infants. GDM complicates 10%–30% of pregnancies, depending on the population screened, strategy undertaken, and diagnostic criteria applied [57]. Early pregnancy detection could potentially reduce adverse maternal and child outcomes in the short- and long-term, and alter intergenerational cardiometabolic trajectories. Clinical risk factor-screening and multivariable modelling each detect ~75% of women with GDM (at SPR ≈ 40%) [58]. However, multivariable modelling detects almost 90% of those who will require insulin and 80% who will require metformin [59]; the 40% SPR could be halved with addition of metabolomics (e.g., lipids) [60]. Unfortunately, negative likelihood ratios >0.20 emphasise that it is not yet possible to predict who will *not* get GDM, or GDM requiring therapy [59].

## Preterm birth

PTB (~8% of pregnancies [61]) is the leading cause of neonatal mortality and morbidity, particularly at <32 weeks [28,29]. Those at highest risk of PTB (those with prior spontaneous preterm birth, cervical insufficiency, or structural uterine abnormalities) are referred to preterm birth clinics for cervical length monitoring, vaginal progesterone, and consideration for cervical cerclage. However, general screening is also needed, because 85% of PTB occurs among the 97% of pregnancies without the strongest clinical risk factors [62].

As PTB prediction in early pregnancy detects no more than 40% of those at risk (at SPR = 10%) [63], PTB prediction relies on risk assessment at 20 weeks, a cost-effective strategy [64]. The risk of very PTB can be derived from previous obstetric history and cervical length measurement at 20–24 weeks [65], with detection rates of ~80% for birth at <28 weeks, 60% at 28–30 weeks, and 50% at 31–33 weeks (at SPR = 10%). Future directions include AI-assisted cervical length measurement in the first trimester [66], and use of AI to integrate large quantities of multiomics data with clinical variables, with early studies suggesting improved predictive accuracy compared with conventional models [67].

## Long-term maternal cardio-renal-metabolic risk

National and international guidelines focus on postnatal follow-up for women following specific pregnancy complications, particularly pre-eclampsia [68] or GDM [69], to prevent maternal cardio-renal-metabolic disease, the leading cause of female death [70]. While robust evidence links pre-eclampsia (and gestational hypertension) with cardio-renal-metabolic outcomes, it is likely this risk is driven primarily by shared underlying risk factors [71–74] which may be used for prediction, such as for chronic hypertension [75]. As prediction and prevention of pregnancy complications improve, removing the gestational ‘signal of risk’ (e.g., pre-eclampsia) that drives postnatal care pathways will make more important a focus on early pregnancy risk factors for cardio-renal-metabolic disease.

## Conclusions and future directions

The inverted pyramid of care describes a focus on risk prediction, prevention and enhanced surveillance for all women from early pregnancy to complement the timely diagnosis and treatment of routine antenatal care. Since the introduction of this theory in 2011, screening has been broadened to include contingent screening at multiple timepoints, and screening later in pregnancy for optimal prediction.

Health systems must evaluate what is affordable, but an advantage of multivariable modelling is that the SPR can be adjusted to accommodate local circumstances. Some of the advances discussed herein are ready for implementation, including: early diagnosis (moving on from prediction) of fetal aneuploidy using cfDNA; early diagnosis of vasa praevia and placental accreta spectrum using ultrasound; prediction (at 11–13 weeks’ gestation) of preterm pre-eclampsia; prediction (at 35–36 weeks’ gestation) of term pre-eclampsia; and mid-pregnancy screening for PTB risk. Promising advances not yet policy-ready include: early pregnancy contingent screening to reduce complexity and cost for prediction of preterm pre-eclampsia; early pregnancy prediction of GDM requiring medical therapy; AI-assisted fetal biometry for prediction of SGA and LGA infants; and long-term prediction of maternal cardio-renal-metabolic disease based on early pregnancy risk factors.

Screening performance will no doubt improve with AI-assisted ultrasound image processing, use of electronic health records, widespread availability of biomarkers, and improvements in multivariable prediction models. This is likely to support de-implementation of low-value screening practices, such as symphysis-fundal height measurement for fetal size, making human and financial resources for health available for better use.

Prediction must expand. For example, venous thromboembolism (1–2 in 1,000 pregnancies) is a leading cause of maternal mortality [76]. While many risk factors are identifiable in early pregnancy, no multivariable model exists to integrate them and guide thromboprophylaxis, an unpleasant and expensive preventive therapy.

Prediction must also progress. Regarding risk as a continuum, and not categorically as high or low, will improve predictive power. Dynamic modelling that incorporates change over time (rather than only at discrete time points) will be facilitated by serial data available in electronic health records, ensuring models work across patient groups, social complexity, and healthcare models.

The ‘inverted pyramid of care’ has progressed since its introduction in 2011. Momentum continues to build for prediction, prevention, personalisation, and participation in maternal and fetal care.

## Abbreviations

AI: artificial intelligence
EFW: estimated fetal weight
FMF: Fetal Medicine Foundation
GDM: Gestational diabetes mellitus
LGA: large-for-gestational age
MAP: mean arterial pressure
PAS: Placenta accreta spectrum
PlGF: placental growth factor
SGA: small-for-gestational age
